# Tipping the scale: the role of a national nutritional supplementation programme for pregnant mothers in reducing low birth weight and neonatal mortality in India

**DOI:** 10.1017/S0007114521000982

**Published:** 2022-01-28

**Authors:** Rajesh Kumar Rai, Sandhya S. Kumar, Devraj J. Parasannanavar, Shweta Khandelwal, Hemalatha Rajkumar

**Affiliations:** 1 Society for Health and Demographic Surveillance, Suri 731101, WB, India; 2 Department of Global Health and Population, Harvard T H Chan School of Public Health, Boston, MA 02115, USA; 3 Department of Economics, University of Göttingen, Göttingen 37073, Germany; 4 Centre for Modern Indian Studies, University of Göttingen, Göttingen 37073, Germany; 5 World Vegetable Center–South and Central Asia, Hyderabad 502324, Telangana, India; 6 Division of Clinical Epidemiology, Indian Council of Medical Research–National Institute of Nutrition, Hyderabad 500007, Telangana, India; 7 Department of Nutrition, Public Health Foundation of India, Gurugram 122003, India; 8 Indian Council of Medical Research–National Institute of Nutrition, Hyderabad 500007, Telangana, India

**Keywords:** Nutrition supplement, Public policy, Child health, Maternal and child health, India

## Abstract

With over 1·3 million *Anganwadi* centres (AWC) (meaning ‘courtyard shelter’), the Indian government runs a nationwide intervention providing nutrition supplement to pregnant mothers to improve the health of their children. Using two successive rounds of the nationally representative cross-sectional National Family Health Survey data (collected during 2005–2006 and 2015–2016) of India, we assessed whether nutrition supplements given to pregnant mothers through AWC were associated with select child health indicators – extremely low birth weight (ELBW), very low birth weight (VLBW), low birth weight (LBW) and neonatal mortality (death during day 0–27) stratified by death during day 0–1, day 2–6 and day 7–27. A total of 148 019 children and 205 593 children were eligible for analysing birth weight and neonatal mortality, respectively. OR with 95% CI, estimated from multivariate logistic regression models, suggest that receipt of nutrition supplements was associated with decreased risk of VLBW (OR: 0·73, 95% CI 0·63, 0·83, *P* < 0·001), LBW (OR: 0·92, 95% CI 0·88, 0·96, *P* < 0·001), but not ELBW (OR: 0·80, 95% CI 0·56, 1·15, *P* = 0·226). Women who always received nutrition supplements during their pregnancy saw lower risk of death of their neonates (OR: 0·67, 95% CI 0·61, 0·73, *P* < 0·001), including death on day 0–1 (OR: 0·66, 95% CI 0·58, 0·74, *P* < 0·001), day 2–6 (OR: 0·69, 95% CI 0·58, 0·82, *P* < 0·001) and day 7–27 (OR: 0·68, 95% CI 0·53, 0·87, *P* = 0·002). Therefore, nutritional supplementation to pregnant mothers appears to be helpful in deterring various stages of neonatal mortality, VLBW and LBW, though it might not be effective in mitigating ELBW. Findings were discussed considering possible limitations of the study.

Benefits of maternal nutrition during pregnancy are well documented. A healthy and balanced nutrition among pregnant mothers leads to healthy birth outcomes and, consequently, better offspring health in later life^(1–7)^. In low-and-middle-income countries, inadequate intake of energy, protein, vitamins and minerals to meet maternal and fetal needs is rampant and dietary intake of vegetables, meat, dairy products and fruit is often insufficient to meet these essential requirements^(3–5)^. India is no exception^(8–10)^, where an unacceptably high undernutrition among pregnant women is modelled to be a risk factor for poor birth outcomes^(11–13)^. The Global Burden of Disease estimates highlight an unacceptably high burden of various indicators of undernutrition among women in India. For example: in 2016, Fe-deficiency anaemia accounted for 11 % of all disability among women in India^(14)^. A high burden of Fe-deficiency anaemia among pregnant mothers has severe consequences on the health of their children^(15,16)^, and nutrition supplements could be helpful in treating Fe-deficiency anaemia.

To mitigate the burden of undernutrition among pregnant women, providing nutrition supplements to pregnant mothers is recommended to be a scalable intervention and has positive effects on a range of health outcomes^(4,6,17,18)^. In line with the evidence, the Indian government established *Anganwadi* centres (AWC) (meaning ‘courtyard shelter’) in 1975 as part of the Integrated Child Development Services programme to combat hunger and malnutrition among women and children^(19,20)^. India currently has over 1·3 million operational AWC managed by *Anganwadi* workers, and among their various responsibilities, *Anganwadi* workers encourage pregnant mothers to eat locally available nutritious foods; they provide education and counselling on exclusive breast-feeding practices to mothers and advise them on supplementary food required for the healthy development of their children^(20,21)^. The need of the Integrated Child Development Services programme was confirmed in the 2013 National Food Security Act (NFSA) where distribution of supplementary nutrition to pregnant and lactating mothers was mandated as part of the right to food, and it was instructed that *Anganwadi* workers will continue providing daily ‘meal, free of charge, during pregnancy and six months after the child birth, through the local *Anganwadi*, so as to meet the nutritional standards’ – 2510 kJ of energy content/energy and 18–20 g of protein^(22)^. Empirical study on the effect of NFSA on reduction of undernutrition in India is non-existent, but some preliminary studies indicate that NFSA is unlikely to greatly affect food consumption, and even if it does, it could only make a small dent to reduce undernutrition^(23)^. Pregnant women registered with AWC are supposed to receive hot cooked meals and micronutrient-fortified and energy-dense food as take-home ration across the country. NFSA instructs all states and union territories of India to develop a menu of commonly accepted nutritious food which will provide the required nutrition. However, there is significant variation in the take-home ration provided across states, reflecting how take-home ration is frequently not aligned with Integrated Child Development Services guidelines for micronutrient composition, and a systematic lack of accountability at AWC remains a key barrier to programmatic effectiveness^(24)^.

To our knowledge, no up-to-date studies exist on whether nutritional supplementation provided to pregnant mothers has helped to improve the health of children born to them. Against this knowledge gap, using two successive rounds of the nationally representative cross-sectional National Family Health Survey data (collected during 2005–2006 and 2015–2016) from India, we investigated if nutrition supplement given to pregnant mothers through AWC was associated with select child health indicators – extremely low birth weight (ELBW), very low birth weight (VLBW), low birth weight (LBW) and neonatal mortality (death during day 0–27) stratified by death during day 0–1, day 2–6 and day 7–27. This study’s findings aim to be helpful for guiding national policies on the role of nutrition intervention during pregnancy in improving child health.

## Methods

### Data set

Two successive rounds of India’s Demographic and Health Survey (collected during 2005–2006 and 2015–2016), commonly known as the National Family Health Survey (NFHS), were used^(25,26)^. NFHS2005–2006 (NFHS-3) and NFHS2015–2016 (NFHS-4) are cross-sectional surveys and widely used for programme and policymaking in India^(27)^. NFHS-3 covered twenty-nine states/union territories, whereas NFHS-4 included thirty-six states/union territories. For NFHS-3, 2001 Census of India and, for NFHS-4, 2011 Census of India sampling frame were used to draw the sample for both rural and urban areas using two-stage stratified random sampling. Villages in rural areas and census enumeration blocks in urban areas served as the primary sampling units or clusters. By virtue of their sampling design, estimates from both rounds of surveys are comparable^(28)^. Details of sampling procedures are furnished elsewhere^(25,26)^. Six administrative regions (Andaman and Nicobar Islands, Chandigarh, Dadra and Nagar Haveli, Daman and Diu, Lakshadweep and Puducherry) were not part of NFHS-3^(25)^, leaving twenty-nine common states/union territories covered in both waves of the NFHS, representing over 99·6 % of India’s population. A household response rate of over 95 % was registered in both NFHS rounds.

To analyse birth weight, 148 019 children were eligible for this study – 14 856 children from NFHS-3 and 133 163 children from NFHS-4. The denominator for analysing neonatal mortality was 205 593, consisting of 33 263 samples from NFHS-3 and 172 330 samples from NFHS-4. NFHS records information on birth weight for children born in the 5 years preceding the survey date, and age at death was recorded for children ever born. However, this study includes the record of birth weight or child mortality only for index children, for two reasons – it would minimise recall errors, and information on primary variable and covariates used in this study was collected only for the index birth.

### Outcome events

Two sets of outcome events were investigated. For the first set of events, three child health indicators related to LBW were considered. Following the guideline developed by the WHO, children’s LBW (in kg) was categorised into three groups: LBW with weight of <2·5 kg, VLBW with weight of <1·5 kg and ELBW with weight of <1·0 kg^(29)^. In both rounds of NFHS, women were asked if the children born to them were weighed at birth, and if the response was affirmative, the weight at birth was recorded in kg. Enumerators were advised to record birth weight from the health card when available; otherwise reporting of birth weight was based on mother’s recall which could have been affected by recall errors, and digit preference which would cause heaping^(30)^.

As a second set of outcome events, neonatal mortality (death during day 0–27) stratified by death during day 0–1, day 2–6 and day 7–27 was analysed. The NFHS asked women about the birth and death history of children ever born to them. In case of death of a child, the age at which the child died was reported. Age at death was recorded in days if the child died within the first month of life, in months if the child died between one month and the second birthday or otherwise in years. In this study, death of children during the first 28 d of life (0–27 d) was defined as neonatal mortality. Neonatal mortality was further investigated by age at death: day 0–1, day 2–6 and day 7–27. This categorisation of age of death is critical as neonatal death varies greatly with days^(12)^.

### Primary variable and covariates

As a primary variable of interest, this study assessed if women received nutritional supplementation during their last pregnancy. In NFHS, for their last birth in the 5 years preceding the survey date, eligible mothers were asked: ‘Did you receive any supplementary nutrition from the *Anganwadi* Centre during this pregnancy?’ If the response was affirmative, a follow-up question was posed: ‘During this pregnancy, were you always able to get the supplementary nutrition from the *Anganwadi* Centre?’ Responses were coded into ‘yes, always’ and ‘no’. Using this information, the primary variable of interest was computed into three groups about receipt of supplementary nutrition – never received, received but not always and always received. As nutritional supplementation during pregnancy is crucial, one may expect that women who always received supplements will have higher odds of experiencing favourable health outcomes of children born to them, compared with women who only sometimes received (but not always) supplements.

Select covariates representing socio-economic characteristics are current age group (in completed years) of mother (15–19, 20–29, 30–39 and ≥40), mother’s age at marriage (<17, 18–20, 21–25 and ≥26), education of mother (no or incomplete primary, primary or incomplete secondary, and secondary or higher), sex of child (male and female), birth order (1, 2, 3, 4 and ≥5), place of residence (urban and rural), social group (others, Scheduled Castes, Scheduled Tribes and Other Backward Classes), religion (Hinduism, Islam, Christianity and others), economic group (poorest, poorer, middle, richer and richest) and state of residence (non-high-focus states and high-focus states). In addition, covariates on maternal healthcare service utilisation included number of antenatal care visits (≥4 and <4), institutional delivery (yes and no) and maternal nutrition status represented by BMI of the mother (underweight, optimum, and overweight and obesity). Variable on sources of birth weight data (from written card and from mother’s recall) was used to analyse LBW, and waves of NFHS (2005–2006 and 2015–2016) were used to capture variation of outcome events by time of survey.

Primary education refers to studying ≤8th grade, and secondary education refers to study of 9th–10th grade. For social group, as per the Constitution of India^(31)^, Scheduled Castes, Scheduled Tribes and Other Backward Classes are historically socially and economically disadvantaged populations; the ‘Others’ category represents the population that has historically been relatively more privileged. NFHS provides the variable of wealth index in its data set. The wealth index is calculated using household assets and durables. The method of constructing the wealth index representing economic groups is presented elsewhere^(32)^. Due to both high fertility and high mortality indicators, nine states are regarded as high-focus states in need of special attention; these are Bihar, Chhattisgarh, Jharkhand, Madhya Pradesh, Odisha, Rajasthan, Uttarakhand, Uttar Pradesh and Assam^(33)^. According to the WHO, a BMI of <18·5 kg/m^2^ is considered a measure of underweight, 18·5–22·99 kg/m^2^ as optimum weight and ≥23 kg/m^2^ is labelled as overweight, including obesity, for the Asian population^(34)^.

### Statistical approach

This study used a combination of descriptive and multivariate analyses. To run the analysis, data from both NFHS were pooled to increase the power of the study, totalling 148 019 children to study LBW, and 205 593 children for studying neonatal mortality. Bivariate analysis was run to understand the proportional difference of outcome events – ELBW, VLBW, LBW and neonatal mortality (death at age 0–27 d) – stratified by age at death (day 0–1, day 2–6 and day 7–27). Multivariable logistic regression analyses were conducted for all outcome variables coded in binary terms (0 and 1). For each outcome, four regression models were run. Model I included the primary variable of interest – receipt of nutritional supplementation during pregnancy and waves of the NFHS survey; model II included all variables from model I and variables representing socio-economic characteristics; model III included all variables of model II and variables representing maternal healthcare service utilisation (ANC and delivery care); model IV included all variables from model III and maternal BMI. This four-step modelling helped separate the possible role of other variables while understanding the association between receiving nutritional supplementation during pregnancy and select outcome events. For all the outcomes on birth weight, one additional variable representing sources of birth weight data was adjusted as birth weight reporting could differ between health card and maternal recall^(35)^. Adjusting NFHS year for all the regression models helped in accounting for secular time trend. Before executing the multivariate analysis, the variables included in the model were tested for multicollinearity by estimating the variance inflation factor and all variance inflation factors were <5·0, indicating a low probability of multicollinearity^(36)^.

Recording of birth weight through mother’s recall is likely to have digit preference, often in multiples of 500 g^(35)^ which leads to heaping^(37)^. To check the sensitivity of this, an alternate analysis with alternate definition of ELBW of ≤1·0 kg, VLBW ≤ 1·5 kg and LBW of ≤2·5 kg was run (data not shown separately), hoping the magnitude of negative association of nutritional supplementation received during pregnancy would be smaller than the standard definition of respective categories. Appropriate sample weighting available with the NFHS data set was used, and ‘svy’ suite available to adjust sample weighting with the statistical software Stata, version 14,^(38)^ was used. *P* value (two-tailed) of <0·05 obtained from logistic regression models was included in the Results and Discussion.

### Ethics statement

Prior to conducting the NFHS, ethical approval was obtained by the implementing institute, the International Institute for Population Sciences, from an independent ethics review committee constituted by the Ministry of Health and Family Welfare, Government of India. NFHS data sets are available in the public domain with all participant identifiers removed. Thus, no separate ethical approval was required for this study.

## Results

Table 1 represents prevalence of ELBW, VLBW and LBW, and prevalence of day of neonatal mortality (day 0–1, day 2–6 and day 7–27) and neonatal mortality with 95 % CI by select background characteristics. Overall, the prevalence of ELBW, VLBW and LBW was 0·13 % (95 % CI 0·11, 0·16), 1·29 % (95 % CI 1·21, 1·37) and 17·7 % (95 % CI 17·4, 18·0), respectively. Women who never received nutritional supplementation experienced a higher prevalence of ELBW, VLBW and LBW of their babies. On the other hand, prevalence of neonatal mortality during day 0–1, day 2–6, day 7–27 and neonatal mortality was 1·12 (95 % CI 1·06, 1·18), 0·53 (95 % CI 0·49, 0·57), 0·35 (95 % CI 0·31, 0·38) and 1·99 (95 % CI 1·91, 2·07), respectively. Women who never received nutrition supplements during their pregnancy had higher prevalence of any type of neonatal mortality, compared with women who received nutritional supplementation.

**Table 1:** Prevalence of extremely low birth weight, very low birth weight and low birth weight, and prevalence of timing of neonatal mortality (day 0–1, day 2–6 and day 7–27) and neonatal mortality (day 0–27) by select background characteristics* (Numbers and percentages, 95 % confidence intervals)

	*n*	Extremely low birth weight		Very low birth weight		Low birth weight			Neonatal mortality (day 0–1)		Neonatal mortality (day 2–6)		Neonatal mortality (day 7–27)		Neonatal mortality (day 0–27)	
		%	95 % CI	%	95 % CI	%	95 % CI	*n*	%	95 % CI	%	95 % CI	%	95 % CI	%	95 % CI
Nutrition supplement during pregnancy																
Never received	64 136	0·16	0·12, 0·21	1·53	1·40, 1·68	18·1	17·7, 18·5	102 029	1·30	1·22, 1·40	0·62	0·56, 0·68	0·42	0·38, 0·48	2·34	2·23, 2·47
Received, but not always	13 918	0·14	0·08, 0·24	1·28	1·05, 1·55	16·7	15·9, 17·5	20 061	1·26	1·07, 1·48	0·57	0·46, 0·71	0·34	0·25, 0·45	2·17	1·92, 2·44
Always received	69 965	0·11	0·08, 0·15	1·06	0·97, 1·17	17·5	17·2, 17·9	83 503	0·87	0·79, 0·95	0·41	0·36, 0·46	0·25	0·21, 0·30	1·53	1·43, 1·63
Current age group of mother																
15–19 years	4926	0·18	0·08, 0·37	2·24	1·74, 2·88	22·2	20·7, 23·8	7136	1·66	1·35, 2·05	1·19	0·90, 1·56	0·73	0·54, 1·00	3·58	3·09, 4·15
20–29 years	102 036	0·12	0·10, 0·15	1·28	1·18, 1·38	17·8	17·4, 18·1	136 533	1·05	0·98, 1·12	0·49	0·44, 0·53	0·30	0·26, 0·34	1·83	1·74, 1·93
30–39 years	37 917	0·16	0·10, 0·23	1·13	0·99, 1·29	16·9	16·3, 17·4	55 715	1·11	1·00, 1·23	0·51	0·44, 0·60	0·39	0·33, 0·46	2·01	1·87, 2·17
≥40 years	3140	0·33	0·12, 0·92	1·73	1·21, 2·49	17·3	15·5, 19·3	6209	2·28	1·82, 2·85	0·69	0·48, 0·99	0·59	0·38, 0·92	3·55	2·99, 4·23
Mother’s age at marriage																
<17 years	48 616	0·16	0·13, 0·21	1·42	1·29, 1·56	19·0	18·5, 19·4	77 747	1·31	1·22, 1·42	0·65	0·58, 0·72	0·40	0·35, 0·45	2·36	2·23, 2·49
18–20 years	52 035	0·11	0·07, 0·17	1·30	1·16, 1·46	17·4	16·9, 17·8	70 413	1·06	0·97, 1·16	0·46	0·40, 0·53	0·35	0·29, 0·41	1·87	1·74, 2·00
21–25 years	37 953	0·13	0·09, 0·18	1·06	0·93, 1·20	16·5	16·0, 17·1	46 345	0·87	0·76, 0·98	0·42	0·35, 0·51	0·26	0·21, 0·33	1·55	1·41, 1·71
≥26 years	9415	0·11	0·05, 0·28	1·41	1·03, 1·93	17·3	16·0, 18·6	11 088	0·93	0·72, 1·20	0·35	0·24, 0·52	0·28	0·17, 0·44	1·55	1·28, 1·89
Education of mother																
No or incomplete primary	40 796	0·19	0·14, 0·25	1·65	1·50, 1·82	20·2	19·7, 20·7	76 403	1·55	1·44, 1·67	0·69	0·62, 0·76	0·49	0·43, 0·56	2·73	2·59, 2·88
Primary or incomplete secondary	72 258	0·13	0·10, 0·17	1·26	1·15, 1·38	18·1	17·7, 18·5	91 125	0·98	0·90, 1·06	0·48	0·42, 0·54	0·31	0·26, 0·36	1·76	1·65, 1·88
Secondary or higher	34 965	0·08	0·05, 0·15	0·94	0·79, 1·13	14·2	13·7, 14·8	38 065	0·59	0·50, 0·69	0·32	0·25, 0·40	0·16	0·12, 0·22	1·07	0·95, 1·20
Sex of child																
Male	80 957	0·11	0·08, 0·14	1·19	1·09, 1·29	16·5	16·2, 16·9	111 618	1·19	1·11, 1·27	0·55	0·50, 0·61	0·33	0·28, 0·37	2·07	1·96, 2·18
Female	67 062	0·17	0·13, 0·22	1·41	1·28, 1·54	19·2	18·8, 19·6	93 975	1·03	0·95, 1·12	0·49	0·44, 0·56	0·37	0·32, 0·42	1·90	1·79, 2·01
Birth order																
1	53 602	0·12	0·09, 0·16	1·40	1·26, 1·56	18·4	18·0, 18·9	64 118	1·12	1·02, 1·23	0·64	0·56, 0·73	0·32	0·27, 0·38	2·08	1·95, 2·23
2	51 626	0·11	0·08, 0·17	1·07	0·95, 1·19	16·7	16·3, 17·2	66 328	0·84	0·76, 0·93	0·36	0·31, 0·42	0·26	0·21, 0·32	1·47	1·35, 1·59
3	23 753	0·17	0·11, 0·26	1·30	1·12, 1·51	17·4	16·7, 18·1	35 887	1·08	0·94, 1·23	0·37	0·30, 0·45	0·31	0·24, 0·40	1·76	1·59, 1·95
4	10 432	0·19	0·11, 0·32	1·61	1·33, 1·95	18·9	17·9, 19·9	18 638	1·35	1·16, 1·57	0·64	0·51, 0·81	0·43	0·32, 0·57	2·42	2·16, 2·72
≥5	8606	0·20	0·10, 0·39	1·60	1·30, 1·97	19·3	18·2, 20·4	20 622	1·97	1·74, 2·22	0·89	0·74, 1·07	0·72	0·58, 0·88	3·57	3·26, 3·91
Place of residence																
Urban	45 159	0·12	0·09, 0·18	1·26	1·10, 1·43	16·7	16·2, 17·3	54 913	0·78	0·69, 0·89	0·35	0·29, 0·43	0·24	0·18, 0·30	1·37	1·25, 1·51
Rural	102 860	0·14	0·11, 0·17	1·30	1·21, 1·39	18·2	17·9, 18·5	150 680	1·25	1·18, 1·32	0·59	0·55, 0·64	0·39	0·35, 0·43	2·24	2·14, 2·33
Social group																
Others	33 283	0·17	0·13, 0·22	1·23	1·06, 1·43	16·6	16·0, 17·2	43 253	0·87	0·77, 0·99	0·44	0·36, 0·53	0·30	0·23, 0·38	1·61	1·46, 1·77
Scheduled castes	28 123	0·15	0·10, 0·23	1·44	1·27, 1·64	18·9	18·2, 19·5	39 699	1·34	1·21, 1·48	0·57	0·49, 0·66	0·41	0·34, 0·49	2·32	2·15, 2·50
Scheduled tribes	26 289	0·13	0·06, 0·25	1·15	0·94, 1·41	19·7	18·8, 20·5	40 509	1·00	0·86, 1·16	0·60	0·49, 0·74	0·39	0·30, 0·50	1·99	1·78, 2·22
Other Backward Classes	60 324	0·11	0·08, 0·15	1·27	1·16, 1·39	17·3	16·9, 17·7	82 132	1·16	1·07, 1·25	0·53	0·48, 0·59	0·33	0·29, 0·38	2·02	1·91, 2·14
Religion																
Hinduism	114 588	0·13	0·11, 0·16	1·26	1·17, 1·35	17·9	17·6, 18·2	153 461	1·12	1·06, 1·19	0·55	0·51, 0·60	0·35	0·32, 0·39	2·03	1·94, 2·11
Islam	16 261	0·18	0·12, 0·26	1·47	1·24, 1·73	17·0	16·2, 17·8	26 429	1·19	1·03, 1·37	0·45	0·37, 0·56	0·35	0·28, 0·45	2·00	1·80, 2·22
Christianity	10 253	0·04	0·01, 0·13	1·34	0·73, 2·42	15·8	14·0, 17·8	16 660	0·82	0·55, 1·22	0·16	0·10, 0·28	0·14	0·06, 0·30	1·12	0·82, 1·54
Others	6917	0·09	0·03, 0·31	1·25	0·85, 1·83	16·9	15·5, 18·4	9043	0·92	0·70, 1·22	0·43	0·26, 0·71	0·24	0·15, 0·40	1·59	1·27, 1·99
Economic group																
Poorest	26 658	0·18	0·12, 0·28	1·55	1·37, 1·76	19·8	19·2, 20·4	49 000	1·62	1·48, 1·76	0·77	0·68, 0·87	0·50	0·43, 0·59	2·89	2·71, 3·08
Poorer	29 860	0·14	0·10, 0·21	1·45	1·28, 1·63	18·8	18·2, 19·4	45 363	1·37	1·24, 1·51	0·57	0·49, 0·66	0·40	0·34, 0·49	2·35	2·18, 2·53
Middle	30 877	0·15	0·10, 0·21	1·17	1·02, 1·33	18·0	17·4, 18·6	41 140	1·05	0·93, 1·19	0·53	0·44, 0·63	0·34	0·27, 0·44	1·92	1·75, 2·11
Richer	30 350	0·12	0·08, 0·18	1·34	1·14, 1·58	18·1	17·4, 18·8	36 828	0·79	0·68, 0·92	0·36	0·29, 0·45	0·24	0·18, 0·32	1·39	1·24, 1·55
Richest	30 274	0·09	0·05, 0·16	0·98	0·83, 1·16	14·3	13·7, 14·9	33 262	0·52	0·43, 0·63	0·30	0·24, 0·39	0·17	0·13, 0·23	1·00	0·87, 1·14
State of residence																
Non-high focus	67 788	0·12	0·09, 0·17	1·15	1·03, 1·28	17·2	16·8, 17·7	85 284	0·70	0·62, 0·78	0·36	0·30, 0·42	0·22	0·18, 0·27	1·27	1·17, 1·39
High focus	80 231	0·15	0·12, 0·18	1·46	1·36, 1·56	18·3	18·0, 18·6	120 309	1·46	1·38, 1·55	0·66	0·61, 0·72	0·45	0·41, 0·50	2·58	2·47, 2·69
Number of ANC visit																
≥4	83 700	0·11	0·08, 0·14	1·08	0·99, 1·18	16·7	16·3, 17·0	94 642	0·78	0·71, 0·86	0·41	0·36, 0·46	0·23	0·19, 0·28	1·42	1·33, 1·52
<4	64 319	0·17	0·13, 0·22	1·59	1·45, 1·74	19·3	18·9, 19·7	110 951	1·44	1·35, 1·53	0·64	0·58, 0·70	0·45	0·40, 0·51	2·53	2·41, 2·65
Institutional delivery																
Yes	136 010	0·12	0·09, 0·14	1·23	1·15, 1·32	17·4	17·2, 17·7	149 214	1·03	0·97, 1·10	0·48	0·43, 0·52	0·27	0·24, 0·31	1·78	1·70, 1·87
No	12 009	0·36	0·22, 0·58	1·94	1·62, 2·33	21·2	20·1, 22·3	56 379	1·37	1·24, 1·50	0·67	0·59, 0·77	0·55	0·47, 0·65	2·59	2·42, 2·78
BMI of mother																
Underweight	34 325	0·18	0·13, 0·25	1·49	1·34, 1·65	21·5	21·0, 22·1	51 926	1·04	0·94, 1·16	0·55	0·47, 0·63	0·40	0·34, 0·47	1·99	1·85, 2·14
Optimum	71 331	0·12	0·09, 0·15	1·24	1·13, 1·36	17·3	16·9, 17·7	101 810	1·18	1·10, 1·27	0·58	0·52, 0·64	0·32	0·28, 0·37	2·08	1·97, 2·20
Overweight and obesity	42 363	0·13	0·08, 0·19	1·19	1·04, 1·38	15·2	14·7, 15·7	51 857	1·08	0·98, 1·20	0·41	0·34, 0·48	0·33	0·27, 0·41	1·82	1·68, 1·98
Sources of birth weight data																
From written card	74 038	0·07	0·05, 0·11	0·96	0·86, 1·07	16·5	16·2, 16·9	N/A	N/A		N/A		N/A		N/A	
From mother’s recall	73 981	0·20	0·16, 0·25	1·66	1·54, 1·79	19·1	18·7, 19·4	N/A	N/A		N/A		N/A		N/A	
Waves of NFHS																
2005–2006	14 856	0·32	0·22, 0·47	1·95	1·67, 2·27	21·0	20·2, 21·9	33 263	1·15	1·01, 1·32	0·74	0·63, 0·87	0·55	0·46, 0·67	2·45	2·24, 2·68
2015–2016	133 163	0·11	0·09, 0·14	1·22	1·14, 1·31	17·4	17·1, 17·7	172 330	1·11	1·05, 1·17	0·48	0·44, 0·52	0·30	0·27, 0·34	1·89	1·81, 1·97
Overall	148 019	0·13	0·11, 0·16	1·29	1·21, 1·37	17·7	17·4, 18·0	205 593	1·12	1·06, 1·18	0·53	0·49, 0·57	0·35	0·31, 0·38	1·99	1·91, 2·07

ANC, antenatal care; N/A, not applicable; NFHS, National Family Health Survey.

* All *n* are unweighted.

Prior to running multivariable logistic regression analysis, variance inflation factor was estimated to check the presence of multicollinearity and all estimates were <5·0 indicating low possibility of multicollinearity (data not shown separately). Whether nutritional supplementation to mothers was associated with ELBW, VLBW and LBW was tested in a logistic regression model, presented with OR in Table 2, whereas full modelling results are presented as supplementary material online (online Supplementary Table S1). ELBW was not associated (>0·05) with mothers receiving nutritional supplementation, irrespective of covariates added in the model (model 1 through model IV). Receipt of supplementary nutrition (whether not always/sometimes or always) appears to have helped reduce VLBW and LBW (model II through IV). For neonatal mortality (stratified by day of death), role of nutrition supplement is presented in Table 3, with elaboration of the full models in the online supplementary material (online Supplementary Table S2). As shown in model IV, compared with women who never received nutritional supplementation during their pregnancy, women who always received nutrition supplements were less likely to experience neonatal mortality (OR: 0·67, 95 % CI 0·61, 0·73, *P* < 0·001). Women always receiving supplementation were also less likely to experience death of the infant in various day increments: day 0–1 (OR: 0·66, 95 % CI 0·58, 0·74, *P* < 0·001), day 2–6 (OR: 0·69, 95 % CI 0·58, 0·82, *P* < 0·001) and day 7–27 (OR: 0·68, 95 % CI 0·53, 0·87, *P* = 0·002), and the unadjusted (model I) association also showed similar results. However, women who only sometimes received nutrition supplements had no association (*P* ≥ 0·05) with prevention of neonatal mortality on day 2–6 and day 7–27, whereas a protective association was observed for death on day 0–1 and neonatal mortality for women who sometimes received nutrition supplements during pregnancy.

**Table 2:** Association between receipt of nutrition supplement during pregnancy and extremely low birth weight, very low birth weight and low birth weight (Odds ratio and 95 % confidence intervals)

	Model I*			Model II**			Model III***			Model IV****		
	OR	95 % CI	*P*	OR	95 % CI	*P*	OR	95 % CI	*P*	OR	95 % CI	*P*
Extremely low birth weight												
Never received	1·00			1·00			1·00			1·00		
Received, but not always	0·94	0·48, 1·85	0·850	0·83	0·42, 1·63	0·592	0·83	0·42, 1·62	0·580	0·83	0·42, 1·63	0·587
Always received	0·87	0·58, 1·31	0·513	0·80	0·55, 1·17	0·255	0·80	0·56, 1·15	0·225	0·80	0·56, 1·15	0·226
Very low birth weight												
Never received	1·00			1·00			1·00			1·00		
Received, but not always	0·85	0·68, 1·06	0·150	0·77	0·61, 0·96	0·020	0·77	0·62, 0·97	0·024	0·78	0·62, 0·97	0·025
Always received	0·76	0·66, 0·88	<0·001	0·71	0·62, 0·82	<0·001	0·73	0·63, 0·83	<0·001	0·73	0·63, 0·83	<0·001
Low birth weight												
Never received	1·00			1·00			1·00			1·00		
Received, but not always	0·92	0·86, 0·98	0·014	0·84	0·79, 0·90	<0·001	0·85	0·79, 0·90	<0·001	0·84	0·79, 0·90	<0·001
Always received	1·01	0·97, 1·05	0·754	0·92	0·88, 0·96	<0·001	0·93	0·89, 0·97	<0·001	0·92	0·88, 0·96	<0·001

*P*, level of significance; ANC, antenatal care; NFHS, National Family Health Survey.

* Model is adjusted for receipt of nutrition supplement, sources of birth weight data, and waves of NFHS.

† Model is adjusted for receipt of nutrition supplement, current age group of mother, mother’s age at marriage, education of mother, sex of child, birth order, place of residence, social group, religion, economic group, state of residence, sources of birth weight data and waves of NFHS.

‡ Model is adjusted for receipt of nutrition supplement, current age group of mother, mother’s age at marriage, education of mother, sex of child, birth order, place of residence, social group, religion, economic group, state of residence, number of ANC visit, institutional delivery, sources of birth weight data and waves of NFHS.

§ Model is adjusted for receipt of nutrition supplement, current age group of mother, mother’s age at marriage, education of mother, sex of child, birth order, place of residence, social group, religion, economic group, state of residence, number of ANC visit, institutional delivery, BMI of mother, sources of birth weight data and waves of NFHS.

**Table 3:** Association between receipt of nutrition supplements during pregnancy and timing of neonatal mortality (day 0–1, day 2–6 and day 7–27) and neonatal mortality (day 0–27)(Odds ratio and 95 % confidence intervals)

	Model I*			Model II†			Model III‡			Model IV§		
	OR	95 % CI	*P*	OR	95 % CI	*P*	OR	95 % CI	*P*	OR	95 % CI	*P*
Neonatal mortality (day 0–1)												
Never received	1·00			1·00			1·00			1·00		
Received, but not always	0·95	0·80, 1·13	0·572	0·81	0·68, 0·98	0·027	0·82	0·68, 0·98	0·031	0·82	0·69, 0·99	0·038
Always received	0·65	0·58, 0·73	<0·001	0·64	0·57, 0·72	<0·001	0·65	0·58, 0·73	<0·001	0·66	0·58, 0·74	<0·001
Neonatal mortality (day 2–6)												
Never received	1·00			1·00			1·00			1·00		
Received, but not always	0·98	0·77, 1·25	0·871	0·84	0·65, 1·070	0·163	0·84	0·65, 1·07	0·159	0·84	0·65, 1·07	0·161
Always received	0·72	0·61, 0·85	<0·001	0·69	0·58, 0·82	<0·001	0·68	0·57, 0·82	<0·001	0·69	0·58, 0·82	<0·001
Neonatal mortality (day 7–27)												
Never received	1·00			1·00			1·00			1·00		
Received, but not always	0·87	0·63, 1·20	0·386	0·74	0·53, 1·02	0·066	0·75	0·54, 1·04	0·084	0·76	0·54, 1·05	0·095
Always received	0·67	0·53, 0·85	0·001	0·66	0·52, 0·83	<0·001	0·67	0·53, 0·86	0·002	0·68	0·53, 0·87	0·002
Neonatal mortality (day 0–27)												
Never received	1·00			1·00			1·00			1·00		
Received, but not always	0·94	0·83, 1·08	0·384	0·80	0·70, 0·92	0·002	0·81	0·71, 0·93	0·002	0·81	0·71, 0·93	0·003
Always received	0·67	0·61, 0·73	<0·001	0·65	0·59, 0·71	<0·001	0·66	0·60, 0·72	<0·001	0·67	0·61, 0·73	<0·001

*P*, level of significance; ANC, antenatal care; NFHS, National Family Health Survey.

* Model is adjusted for receipt of nutrition supplement, and waves of NFHS.

† Model is adjusted for receipt of nutrition supplement, current age group of mother, mother’s age at marriage, education of mother, sex of child, birth order, place of residence, social group, religion, economic group, state of residence and waves of NFHS.

‡ Model is adjusted for receipt of nutrition supplement, current age group of mother, mother’s age at marriage, education of mother, sex of child, birth order, place of residence, social group, religion, economic group, state of residence, number of ANC visit, institutional delivery and waves of NFHS.

§ Model is adjusted for receipt of nutrition supplement, current age group of mother, mother’s age at marriage, education of mother, sex of child, birth order, place of residence, social group, religion, economic group, state of residence, number of ANC visit, institutional delivery, BMI of mother and waves of NFHS.

## Discussion

Nutritional supplementation to women during their pregnancy is crucial for health of their children. In India, *Anganwadi* Workers of the AWC under the Integrated Child Development Services programme are given responsibility for distributing nutritional supplementation, primarily in the form of take-home ration, to pregnant mothers. In absence of a nationwide evaluative study, using nationally representative NFHS data, this study assesses whether nutritional supplementation given to women during their last pregnancy was associated with select child health indicators – ELBW, VLBW and LBW, and neonatal mortality as well as day of neonatal mortality (day 0–1, day 2–6 and day 7–27).

Multivariate analysis revealed that receipt of nutrition supplements during pregnancy is not associated (*P* ≥ 0·05) with ELBW. The primary causes of ELBW are pre-term birth and intra-uterine growth restriction, and prevention of pre-term birth and intra-uterine growth restriction is multi-factorial where biological pathways and preventive measures for these two conditions are different^(39)^. But whether the role of nutrition supplements during pregnancy outweighs the biological pathways and preventive measures requires further investigation. On the other hand, multivariable analysis revealed that providing nutritional supplementation (sometimes/not always and always) during pregnancy might be helpful in reducing VLBW and LBW. This finding is consistent with interventions confirming the role of supplementary nutrition in mitigating LBW^(18,40)^, but evidence on the role of supplementary nutrition in VLBW in India is absent, and this study offers some insight into the issue.

In the case of neonatal mortality, women who always received nutrition supplements during their last pregnancy experienced higher odds of survival of their neonates of all ages. However, women who sometimes received nutrition supplement had no association (*P* ≥ 0·05) with prevention of mortality on day 2–6 and day 7–27. These estimates indicate the benefits of systematic and intense nutrition supplement intervention in reducing neonatal mortality^(41)^ and unorganised and untimely intervention of nutritional supplementation may not be useful^(42)^ in preventing mortality on day 2–6 and day 7–27. In India, about 57 % of all neonatal deaths occur in the first 3 d after birth and two-thirds of these deaths occur on the first day, that is, within 24 h of birth^(12)^. Prevention of neonatal mortality would require quality prenatal care including nutrition-sensitive interventions, coupled with prevention strategy of death due to prematurity, malformations and sepsis^(12)^. While nutrition supplements such as Fe-and-folic-acid, Ca and other supplements to pregnant mothers are crucial for mitigating neonatal death, the prevention of overall death also had to do more with quality of emergency obstetric care of pregnant women in health facilities as well as special care to the new-born unit and postnatal care^(12,43)^. Well-organised home-based postnatal care could be useful for preventing neonatal death on day 2–6 and day 7–27.

This study acknowledges certain limitations in light of the findings. First, data on all possible determinants of birth weight and neonatal mortality were not available in the NFHS, thus not included in regression modelling. Second, the most information was self-reported or reported by mothers on behalf of their children, thus reporting might be affected by recall errors and social desirability bias. Third, information on birth weight was based on data from the health card and mother’s recall, which reduced the sample size and may underestimate the association. However, the prevalence of LBW in this study being comparable to the general population offers confidence about generalisability of the study findings. Fourth, supported by the sensitivity analysis (data not shown separately), the multivariable models adjusted for recording of birth weight data indicated that mothers’ recall had higher likelihood to record ELBW, VLBW and LBW (online supplementary Table S1). Fifth, while the association between nutritional supplementation to pregnant mothers and outcome events should be interpreted cautiously as detailed information on nutrition supplements (e.g. consumption pattern, nutritional content, intra-household distribution, preparation methods, etc. among other dimensions of nutrition) were not available with NFHS data. Sixth and finally, the response against receipt of nutritional supplementation from AWC is heavily contingent upon the pregnant mother and her family member’s knowledge, awareness and nutrition supplement-seeking behaviours. Thus, reporting about receipt of supplementation could be variable, and a careful interpretation of study findings is needed. Despite these limitations, with a coverage of 99·6 % of India’s children aged 6–59 months and high external validity (no sample selection bias was recorded), findings of this study successfully demonstrated the importance of nutritional supplementation for pregnant mothers in reducing the burden of various stages of LBW and neonatal mortality in India.

Reduction of LBW at the rate of 2 % per annum has been a prime target of the Prime Minister’s Overarching Scheme for Holistic Nutrition or POSHAN *Abhiyaan*^(44)^. Additionally, the 2017 National Health Policy emphasised the prevention of neonatal mortality by ensuring required nutrition to mothers and children^(45)^. Health has been a state subject in India, dictated by the constitution of India. Thus, central government must encourage every state and union territory to follow NFSA guidelines and strengthen the performance of AWC while exercising appropriate monitoring and evaluation. Pregnant mothers enrolled with AWC should be encouraged to receive and consume nutritional supplementation as directed. Failure to improve child health through the nutritional supplementation programme designed for pregnant mothers would not only compromise the targets set under POSHAN *Abhiyaan* and the National Health Policy, but also erode the foundation of health for coming generations.

## Supplementary material

For supplementary material accompanying this paper visit https://doi.org/10.1017/S0007114521000982. click here to view supplementary material

## References

[1] Chia AR , Chen LW , Lai JS , et al. (2019) Maternal dietary patterns and birth outcomes: a systematic review and meta-analysis. Adv Nutr 10, 685–695. 31041446 10.1093/advances/nmy123 PMC6628847

[2] Stephenson J , Heslehurst N , Hall J , et al. (2018) Before the beginning: nutrition and lifestyle in the preconception period and its importance for future health. Lancet 391, 1830–1841. 29673873 10.1016/S0140-6736(18)30311-8 PMC6075697

[3] World Health Organization (2016) WHO Recommendations on Antenatal Care for a Positive Pregnancy Experience. Geneva: World Health Organization. 28079998

[4] Dean SV , Lassi ZS , Imam AM , et al. (2014) Preconception care: nutritional risks and interventions. Reprod Health 11, S3. 10.1186/1742-4755-11-S3-S3 PMC4196560 25415364

[5] Salam RA , Das JK & Bhutta ZA (2014) Multiple micronutrient supplementation during pregnancy and lactation in low-to-middle-income developing country settings: impact on pregnancy outcome. Ann Nutr Metab 65, 4–12. 25227399 10.1159/000365792

[6] Bhutta ZA , Das JK , Rizvi A , et al. (2013) Evidence-based interventions for improvement of maternal, child nutrition: what can be done, at what cost? Lancet 382, 452–477. 23746776 10.1016/S0140-6736(13)60996-4

[7] Abu-Saad K & Fraser D (2010) Maternal nutrition and birth outcomes. Epidemiol Rev 32, 5–25. 20237078 10.1093/epirev/mxq001

[8] Sharma M , Kishore A , Roy D , et al. (2020) A comparison of the Indian diet with the EAT-Lancet reference diet. BMC Public Health 20, 812. 32471408 10.1186/s12889-020-08951-8 PMC7260780

[9] Shankar B , Agrawal S , Beaudreault AR , et al. (2017) Dietary and nutritional change in India: implications for strategies, policies, and interventions. Ann NY Acad Sci 1395, 49–59. 28306140 10.1111/nyas.13324

[10] Green R , Milner J , Joy EJ , et al. (2016) Dietary patterns in India: a systematic review. Br J Nutr 116, 142–148. 27146890 10.1017/S0007114516001598 PMC4890343

[11] India State Level Disease Burden Initiative Malnutrition Collaborators (2019) The burden of child and maternal malnutrition and trends in its indicators in the states of India: the Global Burden of Disease Study 1990–2017. Lancet Child Adolesc Health 3, 855–870. 31542357 10.1016/S2352-4642(19)30273-1 PMC6839043

[12] Sankar MJ , Neogi SB , Sharma J , et al. (2016) State of newborn health in India. J Perinatol 36, S3–S8. 10.1038/jp.2016.183 PMC5144119 27924104

[13] Ramakrishnan U , Lowe A , Vir S , et al. (2012) Public health interventions, barriers, and opportunities for improving maternal nutrition in India. Food Nutr Bull 33, S71–S92. 22913108 10.1177/15648265120332S105

[14] Indian Council of Medical Research, Public Health Foundation of India & Institute for Health Metrics and Evaluation (2017) India: Health of the Nation’s States—The India State-Level Disease Burden Initiative. New Delhi: ICMR, PHFI, and IHME.

[15] Cappellini MD , Musallam KM & Taher AT (2020) Iron deficiency anaemia revisited. J Intern Med 287, 153–170. 31665543 10.1111/joim.13004

[16] Rai RK , Fawzi WW , Barik A , et al. (2018) The burden of iron-deficiency anaemia among women in India: how have iron and folic acid interventions fared?. WHO South East Asia J Public Health 7, 18–23. 29582845 10.4103/2224-3151.228423

[17] Barker M , Dombrowski SU , Colbourn T , et al. (2018) Intervention strategies to improve nutrition and health behaviours before conception. Lancet 391, 1853–1864. 29673875 10.1016/S0140-6736(18)30313-1 PMC6075694

[18] da Silva Lopes K , Ota E , Shakya P , et al. (2017) Effects of nutrition interventions during pregnancy on low birthweight: an overview of systematic reviews. BMJ Glob Health 2, e000389. 10.1136/bmjgh-2017-000389 PMC5623264 29018583

[19] Singh PK , Dubey R , Singh L , et al. (2020) Public health interventions to improve maternal nutrition during pregnancy: a nationally representative study of iron and folic acid consumption and food supplements in India. Public Health Nutr 23, 2671–2686. 32605672 10.1017/S1368980020001007 PMC10200584

[20] Kumar S & Rai RK (2015) Role of India’s anganwadi center in securing food and nutrition for mothers and children. J Agr Food Inform 16, 174–182.

[21] NITI Aayog (2015) A Quick Evaluation Study of Anganwadis under ICDS. Programme Evaluation Organisation. New Delhi: Government of India.

[22] Ministry of Law and Justice (2013) The National Food Security Act 2013. Ministry of Law and Justice. New Delhi: Government of India.

[23] Kjelsrud A & Somanathan R (2017) Malnutrition and the National Food Security Act. Ideas for India. October 31 https://www.ideasforindia.in/topics/macroeconomics/malnutrition-and-the-national-food-security-act.html (accessed February 2021).

[24] Sight and Life Foundation (2020) Take–home rations: a compendium. Bangalore: Sight and Life Foundation. https://www.wcdsbp.org/publications/THR-Compendium_220720.pdf (accessed February 2021).

[25] International Institute for Population Sciences (IIPS) & ICF (2007) National Family Health Survey (NFHS-3), 2005–2006. Mumbai: IIPS.

[26] International Institute for Population Sciences (IIPS) & ICF (2017) National Family Health Survey (NFHS-4), 2015–2016. Mumbai: IIPS.

[27] Dandona R , Pandey A & Dandona L (2016) A review of national health surveys in India. Bull World Health Organ 94, 286–296A. 27034522 10.2471/BLT.15.158493 PMC4794301

[28] Corsi DJ , Neuman M , Finlay JE , et al. (2012) Demographic and health surveys: a profile. Int J Epidemiol 41, 1602–1613. 23148108 10.1093/ije/dys184

[29] United Nations Children’s Fund, World Health Organization (2004) Low Birthweight: Country, Regional and Global Estimates. New York: UNICEF.

[30] Swaminathan A , Kim R & Subramanian SV (2020) Association does not imply prediction: the accuracy of birthweight in predicting child mortality and anthropometric failure. Ann Epidemiol 50, 7–14. 32795601 10.1016/j.annepidem.2020.08.001

[31] Ministry of Personnel, Public Grievances & Pensions (2016) Reservation for SC/ST and OBC. Press Information Bureau, Government of India. July 20 https://pib.gov.in/newsite/PrintRelease.aspx?relid=147326 (accessed February 2021).

[32] Rutstein SO & Johnson K (2004) The DHS Wealth Index. DHS Comparative Reports No. 6. Calverton, MD: ORC Macro.

[33] Kumar C , Singh PK & Rai RK (2012) Under-five mortality in high focus states in India: a district level geospatial analysis. PLoS One 7, e37515. 22629412 10.1371/journal.pone.0037515 PMC3356406

[34] Expert Consultation WHO (2004) Appropriate body-mass index for Asian populations and its implications for policy and intervention strategies. Lancet 363, 157–163. 14726171 10.1016/S0140-6736(03)15268-3

[35] Balarajan Y , Subramanian SV & Fawzi WW (2013) Maternal iron and folic acid supplementation is associated with lower risk of low birthweight in India. J Nutr 143, 1309–1315. 23761647 10.3945/jn.112.172015

[36] Chatterjee S & Hadi AS (2012) Regression Analysis by Example. 5th ed. Hoboken, NJ: Wiley.

[37] Channon AAR , Padmadas SS & McDonald JW (2011) Measuring birthweight in developing countries: does the method of reporting in retrospective surveys matter? Matern Child Health J 15, 12–18. 20063179 10.1007/s10995-009-0553-3

[38] StataCorp (2015) Stata Statistical Software: Release 14. College Station, TX: StataCorp LP.

[39] Cutland CL , Lackritz EM , Mallett-Moore T , et al. (2017) Low birthweight: case definition & guidelines for data collection, analysis, and presentation of maternal immunization safety data. Vaccine 35, 6492–6500. 29150054 10.1016/j.vaccine.2017.01.049 PMC5710991

[40] Ramakrishnan U (2004) Nutrition and low birthweight: from research to practice. Am J Clin Nutr 79, 17–21. 14684392 10.1093/ajcn/79.1.17

[41] Costello AM & Osrin D (2003) Micronutrient status during pregnancy and outcomes for newborn infants in developing countries. J Nutr 133, 1757S–1764S. 12730495 10.1093/jn/133.5.1757S

[42] World Health Organization (2013) Essential Nutrition Actions: Improving Maternal, Newborn, Infant and Young Child Health and Nutrition. Geneva: World Health Organization. 25473713

[43] Sankar MJ , Natarajan CK , Das RR , et al. (2016) When do newborns die? A systematic review of timing of overall and cause-specific neonatal deaths in developing countries. J Perinatol 36, S1–S11. 10.1038/jp.2016.27 PMC4848744 27109087

[44] Ministry of Women and Child Development (2020) Poshan Abhiyaan. Press Information Bureau, Government of India. March 06 - https://pib.gov.in/newsite/PrintRelease.aspx?relid=199916 (accessed February 2021).

[45] Ministry of Health and Family Welfare (2017) National Health Policy 2017. New Delhi: Ministry of Health and Family Welfare, Government of India.

