# Supplementary material for: Tipping the scale: the role of a national nutritional supplementation programme for pregnant mothers in reducing low birth weight and neonatal mortality in India
Source: Br J Nutr. 2021 Mar 22;127(2):289–97. doi: 10.1017/S0007114521000982 (PMC8756072; doi:10.1017/S0007114521000982)
Supplement: Supplementary file 1 [file S0007114521000982sup001.pdf]

Tipping the scale: the role of a national nutritional supplementation programme for pregnant mothers in reducing low birthweight and neonatal mortality in India

Rai et al.

Supplementary materials

Table S1. Association between receipt of nutrition supplement during pregnancy and extremely low birthweight, very low birthweight, and low birthweight.

	Extremely low birthweight							
	Model I		Model II		Model III		Model IV	
	OR (95% CI)	p	OR (95% CI)	p	OR (95% CI)	p	OR (95% CI)	p
**Nutrition supplement during pregnancy**								
Never received	1.00		1.00		1.00		1.00	
Received, but not always	0.94 (0.48-1.85)	0.850	0.83 (0.42-1.63)	0.592	0.83 (0.42-1.62)	0.580	0.83 (0.42-1.63)	0.587
Always received	0.87 (0.58-1.31)	0.513	0.80 (0.55-1.17)	0.255	0.80 (0.56-1.15)	0.225	0.80 (0.56-1.15)	0.226
**Current age-group of mother**								
15-19			1.00		1.00		1.00	
20-29			0.93 (0.40-2.17)	0.875	0.94 (0.41-2.18)	0.892	0.94 (0.41-2.18)	0.887
30-39			1.24 (0.45-3.41)	0.677	1.28 (0.46-3.56)	0.638	1.26 (0.45-3.55)	0.659
≥40			2.44 (0.51-11.60)	0.261	2.52 (0.53-12.03)	0.248	2.45 (0.50-11.99)	0.269
**Mother's age at marriage**								
<17			1.00		1.00		1.00	
18-20			0.81 (0.50-1.30)	0.375	0.83 (0.51-1.34)	0.448	0.83 (0.51-1.34)	0.448
21-25			1.07 (0.58-1.95)	0.831	1.11 (0.61-2.03)	0.735	1.11 (0.61-2.03)	0.734
≥26			0.86 (0.27-2.73)	0.800	0.90 (0.28-2.86)	0.857	0.90 (0.28-2.86)	0.857
**Education of mother**								
No or incomplete primary			1.00		1.00		1.00	
Primary or incomplete secondary			0.89 (0.55-1.43)	0.620	0.94 (0.58-1.52)	0.791	0.94 (0.58-1.52)	0.792
Secondary or higher			0.70 (0.34-1.45)	0.340	0.75 (0.36-1.58)	0.453	0.75 (0.36-1.57)	0.448
**Sex of child**								
Male			1.00		1.00		1.00	
Female			1.56 (1.07-2.28)	0.021	1.56 (1.07-2.28)	0.022	1.56 (1.07-2.28)	0.022
**Birth order**								
1			1.00		1.00		1.00	
2			0.90 (0.55-1.48)	0.681	0.86 (0.53-1.40)	0.546	0.86 (0.53-1.39)	0.529
3			1.07 (0.59-1.94)	0.818	0.98 (0.54-1.78)	0.955	0.98 (0.54-1.78)	0.954
4			0.99 (0.48-2.02)	0.970	0.88 (0.43-1.81)	0.731	0.88 (0.43-1.80)	0.720
≥5			0.74 (0.27-2.01)	0.552	0.65 (0.24-1.77)	0.397	0.65 (0.24-1.78)	0.403
**Place of residence**								
Urban			1.00		1.00		1.00	
Rural			0.93 (0.61-1.43)	0.745	0.90 (0.58-1.40)	0.645	0.90 (0.59-1.40)	0.650
**Social group**								
Others			1.00		1.00		1.00	
Scheduled castes			0.88 (0.53-1.47)	0.619	0.88 (0.53-1.47)	0.629	0.88 (0.53-1.48)	0.635

Scheduled tribes			0.71 (0.31-1.60)	0.405	0.64 (0.28-1.48)	0.301	0.64 (0.28-1.48)	0.299
Other Backward Classes			0.63 (0.38-1.04)	0.073	0.64 (0.39-1.06)	0.081	0.64 (0.39-1.06)	0.081
**Religion**								
Hinduism			1.00		1.00		1.00	
Islam			1.27 (0.83-1.96)	0.271	1.22 (0.79-1.87)	0.365	1.22 (0.79-1.87)	0.370
Christianity			0.30 (0.07-1.21)	0.091	0.30 (0.07-1.22)	0.092	0.30 (0.07-1.23)	0.095
Others			0.73 (0.20-2.66)	0.639	0.70 (0.19-2.56)	0.589	0.70 (0.19-2.57)	0.595
**Economic group**								
Poorest			1.00		1.00		1.00	
Poorer			0.83 (0.44-1.58)	0.579	0.88 (0.47-1.67)	0.701	0.88 (0.46-1.67)	0.698
Middle			0.85 (0.43-1.72)	0.657	0.95 (0.47-1.90)	0.882	0.95 (0.47-1.91)	0.886
Richer			0.63 (0.28-1.41)	0.262	0.73 (0.33-1.60)	0.428	0.73 (0.33-1.61)	0.431
Richest			0.42 (0.18-1.00)	0.051	0.50 (0.20-1.25)	0.138	0.50 (0.20-1.25)	0.137
**State of residence**								
Non-high focus			1.00		1.00		1.00	
High Focus			1.03 (0.63-1.70)	0.897	0.97 (0.57-1.65)	0.900	0.98 (0.58-1.66)	0.937
**Number of ANC visit**								
≥4					1.00		1.00	
<4					1.34 (0.78-2.31)	0.292	1.34 (0.78-2.32)	0.288
**Institutional delivery**								
Yes					1.00		1.00	
No					2.36 (1.32-4.25)	0.004	2.37 (1.32-4.26)	0.004
**BMI of mother**								
Underweight							1.37 (0.90-2.09)	0.148
Optimum							1.00	
Overweight and obesity							1.31 (0.79-2.18)	0.297
**Sources of birthweight data**								
From written card	1.00		1.00		1.00		1.00	
From mother’s recall	2.41 (1.56-3.71)	<0.001	2.32 (1.49-3.60)	<0.001	2.32 (1.50-3.60)	<0.001	2.32 (1.50-3.60)	<0.001
**Waves of NFHS**								
2005-06	1.00		1.00		1.00		1.00	
2015-16	0.51 (0.31-0.85)	0.009	0.48 (0.28-0.81)	0.007	0.53 (0.31-0.90)	0.018	0.53 (0.31-0.91)	0.020

	**Very low birthweight**							
	Model I		Model II		Model III		Model IV	
	OR (95% CI)	p	OR (95% CI)	p	OR (95% CI)	p	OR (95% CI)	p
**Nutrition supplement during pregnancy**								
Never received	1.00		1.00		1.00		1.00	
Received, but not always	0.85 (0.68-1.06)	0.150	0.77 (0.61-0.96)	0.020	0.77 (0.62-0.97)	0.024	0.78 (0.62-0.97)	0.025

Always received	0.76 (0.66-0.88)	<0.001	0.71 (0.62-0.82)	<0.001	0.73 (0.63-0.83)	<0.001	0.73 (0.63-0.83)	<0.001
**Current age-group of mother**								
15-19			1.00		1.00		1.00	
20-29			0.63 (0.46-0.86)	0.004	0.64 (0.46-0.87)	0.004	0.63 (0.46-0.87)	0.004
30-39			0.50 (0.34-0.74)	<0.001	0.51 (0.34-0.75)	0.001	0.50 (0.34-0.75)	0.001
≥40			0.62 (0.35-1.09)	0.098	0.63 (0.36-1.11)	0.108	0.62 (0.35-1.09)	0.099
**Mother's age at marriage**								
<17			1.00		1.00		1.00	
18-20			1.09 (0.93-1.27)	0.308	1.10 (0.94-1.28)	0.258	1.10 (0.94-1.28)	0.256
21-25			1.02 (0.83-1.26)	0.830	1.04 (0.84-1.28)	0.726	1.04 (0.84-1.28)	0.724
≥26			1.58 (1.03-2.41)	0.036	1.60 (1.05-2.45)	0.030	1.60 (1.05-2.45)	0.030
**Education of mother**								
No or incomplete primary			1.00		1.00		1.00	
Primary or incomplete secondary			0.82 (0.70-0.96)	0.012	0.84 (0.72-0.98)	0.030	0.84 (0.72-0.98)	0.030
Secondary or higher			0.63 (0.48-0.82)	0.001	0.65 (0.50-0.85)	0.002	0.65 (0.50-0.85)	0.001
**Sex of child**								
Male			1.00		1.00		1.00	
Female			1.18 (1.04-1.35)	0.010	1.18 (1.04-1.35)	0.011	1.18 (1.04-1.35)	0.011
**Birth order**								
1			1.00		1.00		1.00	
2			0.79 (0.67-0.94)	0.009	0.78 (0.65-0.93)	0.005	0.78 (0.65-0.92)	0.005
3			0.90 (0.72-1.11)	0.312	0.86 (0.70-1.07)	0.176	0.86 (0.70-1.07)	0.174
4			1.06 (0.81-1.38)	0.677	1.00 (0.77-1.32)	0.974	1.00 (0.77-1.31)	0.984
≥5			0.97 (0.70-1.33)	0.842	0.91 (0.66-1.25)	0.551	0.91 (0.66-1.26)	0.564
**Place of residence**								
Urban			1.00		1.00		1.00	
Rural			0.93 (0.78-1.11)	0.415	0.91 (0.77-1.09)	0.316	0.92 (0.77-1.09)	0.319
**Social group**								
Others			1.00		1.00		1.00	
Scheduled castes			1.13 (0.92-1.40)	0.249	1.13 (0.91-1.40)	0.260	1.13 (0.91-1.40)	0.255
Scheduled tribes			0.85 (0.64-1.13)	0.270	0.83 (0.62-1.10)	0.201	0.83 (0.63-1.10)	0.201
Other Backward Classes			1.00 (0.83-1.20)	0.990	1.00 (0.83-1.20)	1.000	1.00 (0.83-1.20)	0.990
**Religion**								
Hinduism			1.00		1.00		1.00	
Islam			1.08 (0.88-1.32)	0.457	1.07 (0.87-1.30)	0.532	1.06 (0.87-1.30)	0.544
Christianity			1.26 (0.67-2.37)	0.478	1.25 (0.66-2.37)	0.484	1.26 (0.67-2.38)	0.476
Others			1.10 (0.75-1.61)	0.635	1.08 (0.74-1.59)	0.693	1.08 (0.74-1.59)	0.686
**Economic group**								
Poorest			1.00		1.00		1.00	

Poorer			1.01 (0.83-1.21)	0.955	1.04 (0.86-1.25)	0.714	1.03 (0.86-1.25)	0.720
Middle			0.83 (0.67-1.04)	0.101	0.88 (0.71-1.09)	0.241	0.88 (0.70-1.09)	0.245
Richer			0.97 (0.76-1.24)	0.807	1.04 (0.81-1.33)	0.775	1.03 (0.81-1.33)	0.788
Richest			0.70 (0.51-0.96)	0.028	0.77 (0.56-1.05)	0.093	0.76 (0.55-1.05)	0.093
**State of residence**								
Non-high focus			1.00		1.00		1.00	
High Focus			1.12 (0.97-1.30)	0.131	1.05 (0.89-1.23)	0.565	1.05 (0.90-1.24)	0.518
**Number of ANC visit**								
≥4					1.00		1.00	
<4					1.29 (1.09-1.52)	0.003	1.29 (1.09-1.52)	0.003
**Institutional delivery**								
Yes					1.00		1.00	
No					1.41 (1.15-1.73)	0.001	1.41 (1.15-1.73)	0.001
**BMI of mother**								
Underweight							1.13 (0.98-1.30)	0.085
Optimum							1.00	
Overweight and obesity							1.12 (0.94-1.32)	0.211
**Sources of birthweight data**								
From written card	1.00		1.00		1.00		1.00	
From mother's recall	1.63 (1.42-1.88)	<0.001	1.58 (1.38-1.82)	<0.001	1.58 (1.38-1.82)	<0.001	1.58 (1.38-1.82)	<0.001
**Waves of NFHS**								
2005-06	1.00		1.00		1.00		1.00	
2015-16	0.82 (0.68-0.98)	0.033	0.80 (0.66-0.98)	0.030	0.82 (0.67-1.00)	0.048	0.82 (0.67-1.00)	0.049

	**Low birthweight**							
	Model I		Model II		Model III		Model IV	
	OR (95% CI)	p	OR (95% CI)	p	OR (95% CI)	p	OR (95% CI)	p
**Nutrition supplement during pregnancy**								
Never received	1.00		1.00		1.00		1.00	
Received, but not always	0.92 (0.86-0.98)	0.014	0.84 (0.79-0.90)	<0.001	0.85 (0.79-0.90)	<0.001	0.84 (0.79-0.90)	<0.001
Always received	1.01 (0.97-1.05)	0.754	0.92 (0.88-0.96)	<0.001	0.93 (0.89-0.97)	<0.001	0.92 (0.88-0.96)	<0.001
**Current age-group of mother**								
15-19			1.00		1.00		1.00	
20-29			0.86 (0.78-0.96)	0.005	0.87 (0.78-0.96)	0.005	0.88 (0.80-0.97)	0.013
30-39			0.82 (0.73-0.92)	0.001	0.82 (0.73-0.93)	0.001	0.86 (0.76-0.96)	0.009
≥40			0.76 (0.63-0.91)	0.004	0.76 (0.64-0.92)	0.004	0.80 (0.66-0.96)	0.017
**Mother's age at marriage**								
<17			1.00		1.00		1.00	
18-20			0.98 (0.94-1.03)	0.388	0.98 (0.94-1.03)	0.472	0.98 (0.94-1.03)	0.419

21-25	1.02 (0.96-1.08)	0.608	1.02 (0.96-1.08)	0.484	1.03 (0.97-1.09)	0.389
≥26	1.15 (1.03-1.28)	0.013	1.16 (1.04-1.29)	0.010	1.16 (1.04-1.30)	0.008
**Education of mother**						
No or incomplete primary	1.00		1.00		1.00	
Primary or incomplete secondary	0.89 (0.85-0.93)	<0.001	0.90 (0.86-0.94)	<0.001	0.91 (0.87-0.95)	<0.001
Secondary or higher	0.69 (0.65-0.74)	<0.001	0.70 (0.66-0.75)	<0.001	0.72 (0.67-0.77)	<0.001
**Sex of child**						
Male	1.00		1.00		1.00	
Female	1.20 (1.15-1.24)	<0.001	1.20 (1.15-1.24)	<0.001	1.20 (1.15-1.25)	<0.001
**Birth order**						
1	1.00		1.00		1.00	
2	0.88 (0.84-0.92)	<0.001	0.87 (0.83-0.91)	<0.001	0.87 (0.83-0.92)	<0.001
3	0.87 (0.82-0.92)	<0.001	0.85 (0.80-0.91)	<0.001	0.86 (0.81-0.92)	<0.001
4	0.92 (0.84-1.00)	0.041	0.89 (0.82-0.97)	0.009	0.90 (0.82-0.97)	0.010
≥5	0.92 (0.83-1.01)	0.096	0.89 (0.81-0.98)	0.023	0.90 (0.81-0.99)	0.033
**Place of residence**						
Urban	1.00		1.00		1.00	
Rural	0.99 (0.94-1.04)	0.580	0.98 (0.93-1.03)	0.411	0.97 (0.92-1.02)	0.234
**Social group**						
Others	1.00		1.00		1.00	
Scheduled castes	1.07 (1.01-1.14)	0.029	1.07 (1.01-1.14)	0.032	1.07 (1.00-1.14)	0.039
Scheduled tribes	1.10 (1.02-1.19)	0.013	1.09 (1.01-1.18)	0.022	1.08 (1.00-1.16)	0.063
Other Backward Classes	1.00 (0.95-1.06)	0.874	1.00 (0.95-1.06)	0.900	1.00 (0.95-1.06)	0.975
**Religion**						
Hinduism	1.00		1.00		1.00	
Islam	0.93 (0.87-0.99)	0.023	0.92 (0.86-0.99)	0.016	0.93 (0.87-1.00)	0.035
Christianity	0.88 (0.76-1.03)	0.102	0.88 (0.76-1.02)	0.096	0.90 (0.78-1.05)	0.176
Others	0.98 (0.88-1.09)	0.730	0.97 (0.87-1.09)	0.646	0.98 (0.88-1.09)	0.741
**Economic group**						
Poorest	1.00		1.00		1.00	
Poorer	0.98 (0.92-1.04)	0.455	0.99 (0.94-1.05)	0.788	1.00 (0.95-1.06)	0.879
Middle	0.96 (0.90-1.02)	0.211	0.98 (0.92-1.05)	0.608	1.01 (0.95-1.08)	0.686
Richer	1.00 (0.93-1.07)	0.987	1.03 (0.96-1.11)	0.423	1.08 (1.00-1.16)	0.045
Richest	0.79 (0.73-0.86)	<0.001	0.82 (0.75-0.89)	<0.001	0.87 (0.80-0.95)	0.002
**State of residence**						
Non-high focus	1.00		1.00		1.00	
High Focus	1.01 (0.97-1.06)	0.530	0.98 (0.94-1.03)	0.407	0.98 (0.94-1.02)	0.365
**Number of ANC visit**						
≥4			1.00		1.00	

<4					1.13 (1.08-1.18)	<0.001	1.12 (1.07-1.17)	<0.001
**Institutional delivery**								
Yes					1.00		1.00	
No					1.15 (1.08-1.23)	<0.001	1.14 (1.06-1.22)	<0.001
**BMI of mother**								
Underweight							1.26 (1.20-1.32)	<0.001
Optimum							1.00	
Overweight and obesity							0.93 (0.88-0.97)	0.002
**Sources of birthweight data**								
From written card	1.00		1.00		1.00		1.00	
From mother's recall	1.16 (1.12-1.21)	<0.001	1.15 (1.10-1.20)	<0.001	1.15 (1.10-1.19)	<0.001	1.15 (1.10-1.19)	<0.001
**Waves of NFHS**								
2005-06	1.00		1.00		1.00		1.00	
2015-16	0.84 (0.79-0.89)	<0.001	0.84 (0.79-0.90)	<0.001	0.85 (0.79-0.90)	<0.001	0.87 (0.82-0.93)	<0.001

OR: Odds Ratio, CI: Confidence Interval, p: level of significance, ANC: Antenatal Care, BMI: Body Mass Index, NFHS: national family Health Survey.

Table S2. Association between receipt of nutrition supplement during pregnancy and timing of neonatal mortality (day 0-1, day 2-6, and day 7-27) and neonatal mortality.

	**Neonatal mortality (day 0-1)**							
	Model I		Model II		Model III		Model IV	
	OR (95% CI)	p	OR (95% CI)	p	OR (95% CI)	p	OR (95% CI)	p
**Nutrition supplement during pregnancy**								
Never received	1.00		1.00		1.00		1.00	
Received, but not always	0.95 (0.80-1.13)	0.572	0.81 (0.68-0.98)	0.027	0.82 (0.68-0.98)	0.031	0.82 (0.69-0.99)	0.038
Always received	0.65 (0.58-0.73)	<0.001	0.64 (0.57-0.72)	<0.001	0.65 (0.58-0.73)	<0.001	0.66 (0.58-0.74)	<0.001
**Current age-group of mother**								
15-19			1.00		1.00		1.00	
20-29			0.75 (0.58-0.97)	0.028	0.75 (0.59-0.97)	0.029	0.74 (0.57-0.95)	0.019
30-39			0.67 (0.49-0.90)	0.009	0.67 (0.49-0.91)	0.009	0.64 (0.47-0.86)	0.004
≥40			0.97 (0.65-1.44)	0.865	0.98 (0.65-1.46)	0.905	0.93 (0.62-1.39)	0.713
**Mother's age at marriage**								
<17			1.00		1.00		1.00	
18-20			0.98 (0.86-1.11)	0.706	0.97 (0.86-1.11)	0.684	0.98 (0.86-1.11)	0.716
21-25			0.98 (0.83-1.17)	0.843	0.98 (0.83-1.17)	0.846	0.98 (0.82-1.16)	0.800
≥26			1.18 (0.86-1.62)	0.306	1.18 (0.86-1.62)	0.298	1.17 (0.85-1.60)	0.331
**Education of mother**								
No or incomplete primary			1.00		1.00		1.00	
Primary or incomplete secondary			0.83 (0.73-0.95)	0.007	0.83 (0.73-0.95)	0.007	0.82 (0.72-0.94)	0.004
Secondary or higher			0.59 (0.47-0.73)	<0.001	0.59 (0.47-0.73)	<0.001	0.57 (0.46-0.71)	<0.001
**Sex of child**								
Male			1.00		1.00		1.00	
Female			0.86 (0.77-0.96)	0.005	0.86 (0.77-0.96)	0.005	0.86 (0.77-0.95)	0.005
**Birth order**								
1			1.00		1.00		1.00	
2			0.74 (0.64-0.86)	<0.001	0.74 (0.64-0.86)	<0.001	0.74 (0.64-0.860	<0.001
3			0.78 (0.65-0.94)	0.010	0.79 (0.65-0.95)	0.011	0.78 (0.65-0.94)	0.008
4			0.87 (0.70-1.08)	0.209	0.87 (0.70-1.09)	0.226	0.87 (0.70-1.08)	0.199
≥5			1.12 (0.89-1.41)	0.339	1.12 (0.89-1.42)	0.327	1.11 (0.88-1.40)	0.360
**Place of residence**								
Urban			1.00		1.00		1.00	
Rural			1.07 (0.92-1.25)	0.366	1.07 (0.92-1.25)	0.394	1.09 (0.93-1.27)	0.297
**Social group**								
Others			1.00		1.00		1.00	

Scheduled castes			1.21 (1.01-1.44)	0.038	1.20 (1.01-1.43)	0.042	1.21 (1.01-1.45)	0.034
Scheduled tribes			0.83 (0.67-1.02)	0.077	0.84 (0.68-1.03)	0.099	0.85 (0.69-1.06)	0.147
Other Backward Classes			1.06 (0.91-1.24)	0.439	1.06 (0.91-1.24)	0.468	1.06 (0.91-1.25)	0.434
**Religion**								
Hinduism			1.00		1.00		1.00	
Islam			0.97 (0.82-1.14)	0.673	0.98 (0.83-1.15)	0.772	0.96 (0.82-1.13)	0.647
Christianity			1.09 (0.72-1.65)	0.695	1.09 (0.72-1.66)	0.683	1.06 (0.69-1.61)	0.801
Others			1.21 (0.90-1.62)	0.214	1.21 (0.90-1.63)	0.199	1.20 (0.89-1.62)	0.228
**Economic group**								
Poorest			1.00		1.00		1.00	
Poorer			1.00 (0.87-1.15)	0.969	1.00 (0.87-1.15)	0.981	0.98 (0.85-1.13)	0.797
Middle			0.89 (0.76-1.06)	0.189	0.89 (0.75-1.06)	0.193	0.85 (0.72-1.01)	0.066
Richer			0.75 (0.61-0.92)	0.006	0.75 (0.61-0.93)	0.007	0.70 (0.57-0.87)	0.001
Richest			0.54 (0.42-0.71)	<0.001	0.55 (0.42-0.72)	<0.001	0.50 (0.38-0.65)	<0.001
**State of residence**								
Non-high focus			1.00		1.00		1.00	
High Focus			1.58 (1.37-1.83)	<0.001	1.52 (1.32-1.76)	<0.001	1.54 (1.33-1.78)	<0.001
**Number of ANC visit**								
≥4					1.00		1.00	
<4					1.18 (1.04-1.34)	0.011	1.19 (1.05-1.35)	0.007
**Institutional delivery**								
Yes					1.00		1.00	
No					0.86 (0.75-0.99)	0.041	0.87 (0.76-1.01)	0.059
**BMI of mother**								
Underweight							0.81 (0.72-0.92)	0.001
Optimum							1.00	
Overweight and obesity							1.27 (1.10-1.46)	0.001
**Waves of NFHS**								
2005-06	1.00		1.00		1.00		1.00	
2015-16	1.10 (0.95-1.28)	0.196	1.26 (1.08-1.47)	0.003	1.20 (1.01-1.42)	0.034	1.14 (0.96-1.35)	0.125

	**Neonatal mortality (day 2-6)**							
	Model I		Model II		Model III		Model IV	
	OR (95% CI)	p	OR (95% CI)	p	OR (95% CI)	p	OR (95% CI)	p
**Nutrition supplement during pregnancy**								
Never received	1.00		1.00		1.00		1.00	
Received, but not always	0.98 (0.77-1.25)	0.871	0.84 (0.65-1.070	0.163	0.84 (0.65-1.07)	0.159	0.84 (0.65-1.07)	0.161
Always received	0.72 (0.61-0.85)	<0.001	0.69 (0.58-0.82)	<0.001	0.68 (0.57-0.82)	<0.001	0.69 (0.58-0.82)	<0.001
**Current age-group of mother**								

15-19	1.00		1.00		1.00	
20-29	0.60 (0.43-0.83)	0.002	0.60 (0.43-0.83)	0.002	0.60 (0.43-0.83)	0.002
30-39	0.57 (0.38-0.87)	0.009	0.57 (0.38-0.87)	0.009	0.57 (0.37-0.86)	0.007
≥40	0.54 (0.30-0.96)	0.037	0.54 (0.30-0.97)	0.037	0.54 (0.30-0.96)	0.036
**Mother's age at marriage**						
<17	1.00		1.00		1.00	
18-20	0.91 (0.75-1.09)	0.300	0.90 (0.75-1.09)	0.290	0.91 (0.75-1.09)	0.290
21-25	0.99 (0.78-1.26)	0.925	0.99 (0.77-1.26)	0.914	0.98 (0.77-1.25)	0.899
≥26	0.91 (0.58-1.45)	0.700	0.91 (0.58-1.45)	0.697	0.91 (0.57-1.44)	0.684
**Education of mother**						
No or incomplete primary	1.00		1.00		1.00	
Primary or incomplete secondary	0.92 (0.76-1.12)	0.412	0.92 (0.76-1.11)	0.373	0.91 (0.75-1.11)	0.359
Secondary or higher	0.70 (0.49-0.99)	0.046	0.69 (0.49-0.99)	0.041	0.69 (0.49-0.98)	0.039
**Sex of child**						
Male	1.00		1.00		1.00	
Female	0.88 (0.76-1.03)	0.116	0.88 (0.76-1.03)	0.119	0.88 (0.76-1.03)	0.117
**Birth order**						
1	1.00		1.00		1.00	
2	0.59 (0.47-0.73)	<0.001	0.59 (0.48-0.73)	<0.001	0.59 (0.48-0.73)	<0.001
3	0.51 (0.39-0.66)	<0.001	0.51 (0.39-0.67)	<0.001	0.51 (0.39-0.67)	<0.001
4	0.78 (0.57-1.07)	0.124	0.79 (0.58-1.09)	0.151	0.79 (0.58-1.09)	0.151
≥5	0.98 (0.70-1.38)	0.917	1.00 (0.70-1.42)	0.991	1.00 (0.70-1.42)	0.982
**Place of residence**						
Urban	1.00		1.00		1.00	
Rural	1.19 (0.93-1.52)	0.174	1.19 (0.93-1.53)	0.170	1.19 (0.93-1.53)	0.167
**Social group**						
Others	1.00		1.00		1.00	
Scheduled castes	1.03 (0.79-1.34)	0.850	1.03 (0.79-1.34)	0.840	1.03 (0.79-1.34)	0.834
Scheduled tribes	1.03 (0.75-1.43)	0.846	1.04 (0.75-1.44)	0.807	1.05 (0.76-1.45)	0.784
Other Backward Classes	1.01 (0.80-1.28)	0.921	1.01 (0.80-1.28)	0.918	1.01 (0.80-1.28)	0.919
**Religion**						
Hinduism	1.00		1.00		1.00	
Islam	0.78 (0.61-1.00)	0.047	0.79 (0.61-1.01)	0.056	0.78 (0.61-1.00)	0.054
Christianity	0.38 (0.22-0.67)	0.001	0.38 (0.22-0.68)	0.001	0.38 (0.22-0.66)	0.001
Others	1.04 (0.62-1.75)	0.883	1.05 (0.62-1.76)	0.863	1.04 (0.62-1.75)	0.879
**Economic group**						
Poorest	1.00		1.00		1.00	
Poorer	0.85 (0.69-1.04)	0.106	0.84 (0.69-1.03)	0.096	0.84 (0.68-1.03)	0.086
Middle	0.89 (0.69-1.13)	0.341	0.88 (0.68-1.13)	0.314	0.87 (0.67-1.12)	0.274

Richer			0.68 (0.49-0.94)	0.020	0.67 (0.49-0.93)	0.017	0.66 (0.48-0.91)	0.011
Richest			0.62 (0.39-0.97)	0.037	0.61 (0.38-0.97)	0.036	0.60 (0.38-0.94)	0.026
**State of residence**								
Non-high focus			1.00		1.00		1.00	
High Focus			1.42 (1.17-1.73)	<0.001	1.42 (1.17-1.74)	0.001	1.42 (1.16-1.73)	0.001
**Number of ANC visit**								
≥4					1.00		1.00	
<4					1.03 (0.85-1.24)	0.759	1.03 (0.85-1.25)	0.746
**Institutional delivery**								
Yes					1.00		1.00	
No					0.90 (0.74-1.09)	0.278	0.90 (0.75-1.09)	0.296
**BMI of mother**								
Underweight							0.85 (0.70-1.02)	0.087
Optimum							1.00	
Overweight and obesity							0.97 (0.79-1.19)	0.784
**Waves of NFHS**								
2005-06	1.00		1.00		1.00		1.00	
2015-16	0.72 (0.59-0.86)	0.001	0.83 (0.68-1.01)	0.068	0.80 (0.65-0.99)	0.037	0.79 (0.64-0.97)	0.027

	**Neonatal mortality (day 7-27)**							
	Model I		Model II		Model III		Model IV	
	OR (95% CI)	p	OR (95% CI)	p	OR (95% CI)	p	OR (95% CI)	p
**Nutrition supplement during pregnancy**								
Never received	1.00		1.00		1.00		1.00	
Received, but not always	0.87 (0.63-1.20)	0.386	0.74 (0.53-1.02)	0.066	0.75 (0.54-1.04)	0.084	0.76 (0.54-1.05)	0.095
Always received	0.67 (0.53-0.85)	0.001	0.66 (0.52-0.83)	<0.001	0.67 (0.53-0.86)	0.002	0.68 (0.53-0.87)	0.002
**Current age-group of mother**								
15-19			1.00		1.00		1.00	
20-29			0.44 (0.29-0.66)	<0.001	0.44 (0.29-0.67)	<0.001	0.43 (0.29-0.65)	<0.001
30-39			0.41 (0.24-0.72)	0.002	0.42 (0.24-0.73)	0.002	0.40 (0.23-0.70)	0.001
≥40			0.40 (0.19-0.85)	0.017	0.40 (0.19-0.85)	0.017	0.38 (0.18-0.80)	0.011
**Mother's age at marriage**								
<17			1.00		1.00		1.00	
18-20			1.29 (1.00-1.66)	0.048	1.30 (1.01-1.67)	0.045	1.30 (1.01-1.67)	0.042
21-25			1.29 (0.93-1.79)	0.126	1.30 (0.94-1.80)	0.114	1.30 (0.93-1.79)	0.120
≥26			1.58 (0.86-2.89)	0.138	1.60 (0.87-2.92)	0.130	1.58 (0.86-2.89)	0.139
**Education of mother**								
No or incomplete primary			1.00		1.00		1.00	
Primary or incomplete			0.92 (0.72-1.18)	0.512	0.95 (0.73-1.22)	0.668	0.94 (0.73-1.21)	0.606

secondary						
Secondary or higher	0.58 (0.38-0.89)	0.012	0.60 (0.39-0.92)	0.020	0.59 (0.38-0.90)	0.015
**Sex of child**						
Male	1.00		1.00		1.00	
Female	1.12 (0.93-1.37)	0.239	1.12 (0.92-1.36)	0.249	1.12 (0.92-1.36)	0.255
**Birth order**						
1	1.00		1.00		1.00	
2	0.90 (0.67-1.19)	0.454	0.88 (0.66-1.17)	0.373	0.87 (0.65-1.16)	0.342
3	0.93 (0.64-1.33)	0.677	0.89 (0.62-1.29)	0.545	0.89 (0.61-1.28)	0.518
4	1.14 (0.73-1.76)	0.564	1.09 (0.70-1.69)	0.714	1.08 (0.69-1.67)	0.744
≥5	1.70 (1.07-2.71)	0.025	1.61 (1.01-2.58)	0.047	1.60 (1.00-2.56	0.049
**Place of residence**						
Urban	1.00		1.00		1.00	
Rural	1.11 (0.81-1.52)	0.519	1.09 (0.79-1.50)	0.586	1.11 (0.81-1.52)	0.510
**Social group**						
Others	1.00		1.00		1.00	
Scheduled castes	1.10 (0.77-1.57)	0.586	1.09 (0.77-1.56)	0.621	1.10 (0.77-1.57)	0.588
Scheduled tribes	1.03 (0.69-1.54)	0.881	1.02 (0.68-1.53)	0.931	1.04 (0.69-1.56)	0.857
Other Backward Classes	0.92 (0.68-1.24)	0.587	0.92 (0.68-1.24)	0.565	0.92 (0.68-1.24)	0.592
**Religion**						
Hinduism	1.00		1.00		1.00	
Islam	0.90 (0.65-1.25)	0.531	0.88 (0.64-1.23)	0.463	0.87 (0.63-1.21)	0.412
Christianity	0.49 (0.21-1.11)	0.088	0.47 (0.21-1.09)	0.079	0.47 (0.20-1.08)	0.074
Others	0.91 (0.53-1.55)	0.728	0.90 (0.53-1.53)	0.685	0.89 (0.52-1.51)	0.664
**Economic group**						
Poorest	1.00		1.00		1.00	
Poorer	0.96 (0.74-1.26)	0.775	0.98 (0.75-1.28)	0.888	0.97 (0.74-1.26)	0.797
Middle	0.96 (0.71-1.30)	0.798	1.00 (0.74-1.34)	0.982	0.96 (0.71-1.30)	0.794
Richer	0.75 (0.50-1.13)	0.168	0.79 (0.53-1.18)	0.246	0.74 (0.49-1.12)	0.158
Richest	0.60 (0.37-0.97)	0.037	0.65 (0.41-1.03)	0.069	0.59 (0.36-0.96)	0.032
**State of residence**						
Non-high focus	1.00		1.00		1.00	
High Focus	1.56 (1.22-1.99)	<0.001	1.47 (1.14-1.89)	0.003	1.49 (1.16-1.92)	0.002
**Number of ANC visit**						
≥4			1.00		1.00	
<4			1.14 (0.90-1.45)	0.266	1.16 (0.91-1.46)	0.226
**Institutional delivery**						
Yes			1.00		1.00	
No			1.19 (0.88-1.61)	0.256	1.20 (0.89-1.64)	0.236
**BMI of mother**						

Underweight							1.08 (0.86-1.34)	0.515
Optimum							1.00	
Overweight and obesity							1.50 (1.15-1.95)	0.002
**Waves of NFHS**								
2005-06	1.00		1.00		1.00		1.00	
2015-16	0.62 (0.49-0.78)	<0.001	0.71 (0.56-0.91)	0.006	0.77 (0.59-1.01)	0.059	0.74 (0.57-0.97)	0.031

	**Neonatal mortality**							
	Model I		Model II		Model III		Model IV	
	OR (95% CI)	p	OR (95% CI)	p	OR (95% CI)	p	OR (95% CI)	p
**Nutrition supplement during pregnancy**								
Never received	1.00		1.00		1.00		1.00	
Received, but not always	0.94 (0.83-1.08)	0.384	0.80 (0.70-0.92)	0.002	0.81 (0.71-0.93)	0.002	0.81 (0.71-0.93)	0.003
Always received	0.67 (0.61-0.73)	<0.001	0.65 (0.59-0.71)	<0.001	0.66 (0.60-0.72)	<0.001	0.67 (0.61-0.73)	<0.001
**Current age-group of mother**								
15-19			1.00		1.00		1.00	
20-29			0.63 (0.52-0.75)	<0.001	0.63 (0.52-0.76)	<0.001	0.62 (0.52-0.74)	<0.001
30-39			0.57 (0.46-0.72)	<0.001	0.58 (0.46-0.72)	<0.001	0.55 (0.44-0.69)	<0.001
≥40			0.71 (0.52-0.96)	0.024	0.71 (0.52-0.96)	0.027	0.68 (0.50-0.92)	0.014
**Mother's age at marriage**								
<17			1.00		1.00		1.00	
18-20			1.01 (0.91-1.11)	0.908	1.01 (0.91-1.11)	0.920	1.01 (0.91-1.11)	0.883
21-25			1.03 (0.91-1.18)	0.628	1.03 (0.91-1.18)	0.616	1.03 (0.90-1.17)	0.666
≥26			1.17 (0.92-1.49)	0.212	1.17 (0.92-1.49)	0.202	1.16 (0.91-1.48)	0.233
**Education of mother**								
No or incomplete primary			1.00		1.00		1.00	
Primary or incomplete secondary			0.87 (0.79-0.96)	0.006	0.87 (0.79-0.96)	0.008	0.86 (0.78-0.95)	0.004
Secondary or higher			0.61 (0.51-0.72)	<0.001	0.61 (0.52-0.73)	<0.001	0.60 (0.50-0.71)	<0.001
**Sex of child**								
Male			1.00		1.00		1.00	
Female			0.91 (0.84-0.98)	0.017	0.91 (0.84-0.98)	0.017	0.91 (0.84-0.98)	0.015
**Birth order**								
1			1.00		1.00		1.00	
2			0.71 (0.64-0.80)	<0.001	0.71 (0.64-0.80)	<0.001	0.71 (0.63-0.80)	<0.001
3			0.72 (0.62-0.83)	<0.001	0.72 (0.62-0.83)	<0.001	0.71 (0.62-0.82)	<0.001
4			0.88 (0.75-1.04)	0.137	0.88 (0.74-1.04)	0.135	0.87 (0.74-1.03)	0.117
≥5			1.16 (0.97-1.39)	0.095	1.16 (0.97-1.39)	0.107	1.15 (0.96-1.38)	0.120
**Place of residence**								

Urban			1.00		1.00		1.00	
Rural			1.11 (0.98-1.26)	0.094	1.11 (0.98-1.25)	0.108	1.12 (0.99-1.27)	0.072
**Social group**								
Others			1.00		1.00		1.00	
Scheduled castes			1.14 (0.99-1.31)	0.063	1.14 (0.99-1.31)	0.069	1.14 (1.00-1.31)	0.056
Scheduled tribes			0.92 (0.78-1.08)	0.298	0.92 (0.78-1.09)	0.340	0.94 (0.80-1.11)	0.454
Other Backward Classes			1.02 (0.91-1.16)	0.704	1.02 (0.90-1.15)	0.738	1.02 (0.91-1.16)	0.694
**Religion**								
Hinduism			1.00		1.00		1.00	
Islam			0.90 (0.79-1.03)	0.115	0.91 (0.80-1.03)	0.136	0.90 (0.79-1.02)	0.097
Christianity			0.76 (0.55-1.07)	0.113	0.76 (0.55-1.07)	0.113	0.74 (0.53-1.04)	0.082
Others			1.11 (0.87-1.40)	0.405	1.11 (0.87-1.41)	0.393	1.10 (0.87-1.39)	0.439
**Economic group**								
Poorest			1.00		1.00		1.00	
Poorer			0.95 (0.86-1.06)	0.357	0.95 (0.86-1.06)	0.375	0.94 (0.84-1.04)	0.244
Middle			0.90 (0.80-1.02)	0.111	0.91 (0.80-1.03)	0.129	0.87 (0.77-0.99)	0.040
Richer			0.73 (0.62-0.86)	<0.001	0.73 (0.62-0.86)	<0.001	0.69 (0.59-0.82)	<0.001
Richest			0.57 (0.46-0.70)	<0.001	0.58 (0.47-0.72)	<0.001	0.53 (0.43-0.66)	<0.001
**State of residence**								
Non-high focus			1.00		1.00		1.00	
High Focus			1.54 (1.39-1.71)	<0.001	1.49 (1.34-1.66)	<0.001	1.50 (1.35-1.68)	<0.001
**Number of ANC visit**								
≥4					1.00		1.00	
<4					1.13 (1.03-1.25)	0.011	1.14 (1.04-1.26)	0.007
**Institutional delivery**								
Yes					1.00		1.00	
No					0.92 (0.83-1.03)	0.147	0.93 (0.83-1.04)	0.201
**BMI of mother**								
Underweight							0.86 (0.78-0.95)	0.002
Optimum							1.00	
Overweight and obesity							1.22 (1.10-1.36)	<0.001
**Waves of NFHS**								
2005-06	1.00		1.00		1.00		1.00	
2015-16	0.87 (0.79-0.97)	0.011	1.01 (0.90-1.12)	0.904	0.98 (0.87-1.11)	0.780	0.95 (0.84-1.07)	0.355

OR: Odds Ratio, CI: Confidence Interval, p: level of significance, ANC: Antenatal Care, BMI: Body Mass Index, NFHS: national family Health Survey.
